# Chronic Obstructive Pulmonary Disease Increases the Risk of Hip Fracture: A Nationwide Population-Based Cohort Study

**DOI:** 10.1038/srep23360

**Published:** 2016-03-18

**Authors:** Shih-Wei Huang, Wei-Te Wang, Lin-Chuan Chou, Hung-Chou Chen, Tsan-Hon Liou, Hui-Wen Lin

**Affiliations:** 1 Department of Physical Medicine and Rehabilitation, Shuang Ho Hospital, Taipei Medical University, Taipei, Taiwan; 2 Department of Physical Medicine and Rehabilitation, School of Medicine, College of Medicine, Taipei Medical University, Taipei, Taiwan; 3 Department of Physical Medicine and Rehabilitation, Changhua Christian Hospital, Changhua, Taiwan; 4 Graduate Institute of Injury Prevention, Taipei Medical University, Taipei, Taiwan; 5 Department of Mathematics, Soochow University, Taipei, Taiwan; 6 Evidence-Based Medicine Center, Wan Fang Hospital, Taipei Medical University, Taipei, Taiwan

## Abstract

Hip fractures can lead to functional disability and high mortality rates among elderly patients. The aim of this study was to investigate whether chronic obstructive pulmonary disease (COPD) is a risk factor for hip fracture. A retrospective population-based 4-year cohort study was conducted using case–control matched analysis of data from the Taiwan Longitudinal Health Insurance Database 2005 (LHID2005). Patients with a diagnosis of COPD between January 1, 2004 and December 31, 2007 were enrolled. A 2-stage approach and data from the National Health Interview Survey 2005 were applied to adjust for missing confounders in the LHID2005 cohort. Hazard ratios (HRs) and adjusted HRs were estimated hip fracture risk for the COPD. We enrolled 16,239 patients in the COPD cohort and 48,747 (1:3) patients in non-COPD cohort. The hip fracture incidences were 649 per 100,000 person-years in the study cohort and 369 per 100,000 person-years in non-COPD cohort. The hip fracture HR during the follow-up period was 1.78 (*P* < 0.001) and the adjusted hip fracture HR was 1.57 (*P* < 0.001) after adjustment for covariates by using the 2-stage approach method. Patients with COPD were at hip fracture risk and fracture-prevention strategies are essential for better quality of care.

Chronic obstructive pulmonary disease (COPD) is a slow progressive disease characterized by chronic inflammation of the airways and airflow obstruction. COPD has a prevalence of 5–13% and is the fourth leading cause of death worldwide^1^,^2^. COPD is one of the most prevalent causes of death in North America and by 2020 it will be the third largest disease burden worldwide^3^,^4^. COPD mainly affects pulmonary function, but also affects nonrespiratory systems^5^,^6^. In addition, previous studies have reported muscle strength and exercise capacity deficits among patients with COPD resulting in impairment of postural control and balance function^7^,^8^,^9^,^10^.

Hip fracture can lead to functional disability and high mortality rates in elderly people^11^,^12^. To regain function in independent daily living, nearly all patients with hip fracture require surgery and approximately 50% of patients regain previous mobility^13^. In addition, approximately 25% of patients receive long-term nursing home care despite independently performing daily activities before experiencing hip fracture^14^,^15^. The medical cost incurred because of hip fracture is high, and an economic burden of more than $20 billion was estimated in 1997^16^. Ninety percent hip fractures are caused by falls^17^, and fall injuries are common in patients with COPD^18^. A recent prospective observational study indicated that approximately one third of ambulatory patients with COPD had fall accidents during a 6-month follow-up period. We hypothesized that COPD is associated with hip fracture because fall injury rates are high among patients with COPD.

Falls in patients with COPD could have major complications, including mortality and morbidities such as hip fracture, potentially imposing an economic burden on health care services. Although previous studies have indicated a high fall incidence among patients with COPD, hip fracture risk has not been investigated extensively^19^. A large-scale population-based study analyzing COPD and hip fracture risk is lacking. Moreover, hip fracture prevention is a major public health concern, and identifying hip fracture risk factors is correlated to effective fall strategy establishment. Therefore, we conducted this retrospective population-based cohort study to investigate the hip fracture risk among patients with COPD.

## Methods

### Study design and study population

#### Data source

A retrospective population-based cohort study was conducted using case–control matched analysis. Patient data were obtained from the Taiwan Longitudinal Health Insurance Database 2005 (LHID2005). The National Health Insurance program, established in Taiwan in March 1995, has over 25 million enrollees and covers more than 99% of the population of Taiwan. The LHID2005 contains data on 25.68 million claims from one million beneficiaries randomly sampled from the Registry for Beneficiaries of the National Health Insurance Research Database (NHIRD). The claims files contain information on ambulatory care; inpatient care; pharmacy use; date of service; International Classification of Diseases, Ninth Revision, Clinical Modification (ICD-9-CM) diagnostic codes; and claimed medical expenses. The National Health Research Institutes manages the claims data and provides scrambled random identification numbers for insured patients to protect their privacy. For ethical reasons, the database provides de-identified secondary data. The data were analyzed anonymously and the requirement for informed consent was waived by institution of review board and this study is accordance to the guideline of STROBE (STrengthening the Reporting of OBservational studies in Epidemiology).

#### Study patients

Patients with ambulatory care claims containing ICD-9-CM code 490–496 (COPD) between January 1, 2004 and December 31, 2007 were identified. To ensure that the COPD diagnoses were accurate, patients who received consistent diagnoses according to the ICD-9-CM at least 3 times in outpatient clinics or a primary diagnosis of COPD during hospitalization within 1 year were selected. Initially, 21,341 patients with COPD were enrolled. However, 4880 patients whose data were missing or were aged <50 years and 222 patients with diagnoses of previously sustained hip fracture (ICD-9-CM code 820 and 821) were excluded. The study cohort comprised 16,239 patients with COPD, and the control cohort comprised 48,717 patients without COPD in a 1:3 ratio matched by age and sex in group level. And the patients with previous hip fracture were also excluded in control cohort. Each patient was monitored for 4 years, from the entry date to the diagnosis of hip fracture in an outpatient clinic, primary diagnosis during hospitalization, or the end of 2008.

#### Baseline comorbidities and inhalation medication

Data on baseline variables, namely age, sex, hypertension (ICD-9-CM codes 401–405), hyperlipidemia (ICD-9-CM codes 272.0–272.4), stroke (ICD-9-CM codes 430–438), diabetes mellitus (DM; ICD-9-CM codes 250, 251), and autoimmune diseases (rheumatoid arthritis, RA; ICD-9-CM code 714.0; systemic lupus erythematous, SLE; ICD-9-CM code 710.0), were obtained for all patients. In addition, usage histories of nonsteroidal anti-inflammatory drugs (NSAIDs) and inhalation medications, such as ipratropium bromide administered using a metered-dose inhaler (MDI); fenoterol hydrobromide administered using an MDI, terbutaline sulfate administered using a Turbuhaler, or salbutamol sulfate administered using an MDI; tiotropium administered using a HandiHaler; formoterol administered using a Turbuhaler or salmeterol administered using an MDI; and formoterol/budesonide administered using a Turbuhaler or salmeterol/fluticasone administered using an MDI, were considered.

#### Statistical analysis and 2-stage propensity score calibration

A Cox model was used to calculate an up to 4-year hip fracture hazard function and evaluate the hip fracture risk between the COPD and control cohorts, after adjustment for age, sex, DM, hypertension, hyperlipidemia, stroke, autoimmune diseases (RA, SLE), and inhaled medication and NSAID use.

To adjust for missing confounders, namely smoking, alcohol use, and body mass index (BMI), in the LHID cohort, a 2-stage approach was applied. In previous studies with 2-stage designs, the method for adjusting for missing confounders involved combining samples from the main data and validating data^18^,^19^. According to the inclusion criteria, we identified 350 patients with COPD in the main study (LHID cohort) and 1050 age- and sex-matched participants in the National Health Interview Survey 2005 (NHIS2005) conducted during 2004–2007 (comparison cohort). The comparison cohort had data on BMI, alcohol use, and smoking, which are crucial variables for patients with COPD that were lacking in the LHID cohort. Thus, a 2-stage propensity score matching method described by Stürmer *et al*. was employed to adjust for the missing high-dimensional confounders^20^,^21^,^22^.

Let X denote an indicator variable for COPD. A value 1 is chosen for patients with COPD and 0 otherwise. Let X_c_ be a vector of observed confounders, namely patient age, sex, hypertension, stroke, hyperlipidemia, DM, autoimmune diseases, and inhaled medication and NSAID use. Let U denote an indicator for the missing confounders, smoking, alcohol use, and BMI. The propensity score is defined by the equation PS = Pr (X = 1|X_c_), and the estimated PS and hip fracture association in a Cox proportional hazard model is defined as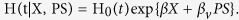 The propensity score 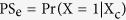 is defined as the error-prone variable and 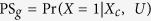 is defined as the gold standard in the validation study. The measurement error model is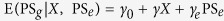, and the regression calibration after adjustment for X_c_ and U confounding factors estimator for the effect of X is 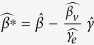
21. The estimated 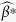 is obtained after adjusting for missing confounders. Lin and Chen proposed TSC statistical methods. Unlike the aforementioned methods, the validity of the TSC method does not rely on a measurement error model^23^. We used the TSC method to estimate beta parameters and obtained results similar to those of the aforementioned process. Furthermore, hip fracture hazard curves based on time-to-event analysis (Kaplan–Meier method) for patients with COPD and the comparison cohort and hip fracture hazard curves stratified by inhaled medications usage for the COPD cohort were plotted. All data analyses were performed using the SAS statistical package (Version 9.4; SAS Institute, Cary, NC, USA). A *P* value of <0.05 was considered statistically significant.

## Results

The COPD cohort in the LHID comprised 16,239 patients with COPD, and the control cohort comprised 48,717 patients without COPD. A total of 350 patients with COPD and 1050 comparison participants without COPD from the NHIS were included in the validation cohort. The demographic characteristics and comorbid medical disorders of these cohorts are shown in Table 1.

The incidence of hip fracture was 649 per 10,000 person-years and 369 per 10,000 person-years in the COPD and control cohorts, respectively. In patients with COPD, the crude hazard ratio (HR) for hip fracture occurrence was 1.78 (95% confidence interval [CI], 1.57–2.01, *P* < 0.001). After adjustment for age, sex, hypertension, stroke, hyperlipidemia and DM, autoimmune diseases, and inhaled medication and NSAID use, the adjusted HR was 1.59 (95% CI, 1.38–1.82, *P* < 0.001). After 2-stage propensity score calibration, the adjusted HR was 1.57 (95% CI, 1.36–1.81, *P* < 0.001) (Table 2).

Table 3 lists the incidence and hazard ratios for hip fracture in COPD patients with inhalation medication (N = 10,362) and without inhalation medication (N = 5,877) in comparison with the control cohort (N = 48,717). The incidence of hip fracture was 717 per 10,000 person-years in the COPD and inhaled medication cohort, whereas it was 595 per 10,000 person-years in the COPD without inhaled medication cohort. The crude HR of hip fracture occurrence for patients with COPD using inhaled medication was 2.04 (95% CI, 1.72–2.41, *P* < 0.001), and the adjusted HR was 1.72 (95% CI, 1.45–2.04, *P* < 0.001). The crude HR for patients with COPD not using inhaled medication was 1.63 (95% CI, 1.40–1.89, *P* < 0.001), and the adjusted HR was 1.46 (95% CI, 1.25–1.69, *P* < 0.001).

**Figure 1:**
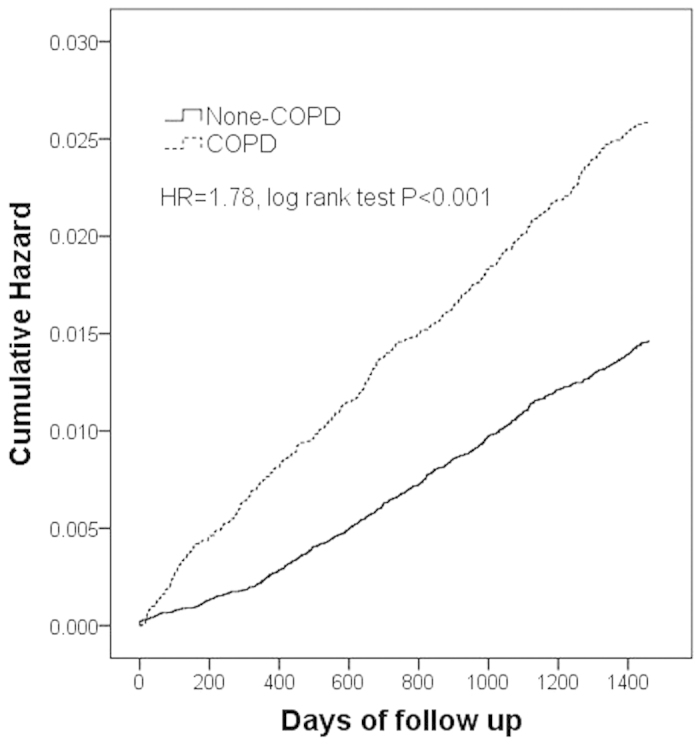
Cumulative hazard rates of hip fracture in chronic obstructive pulmonary disease (COPD) patients and comparison cohort during the 4-year follow-up period.

**Figure 2:**
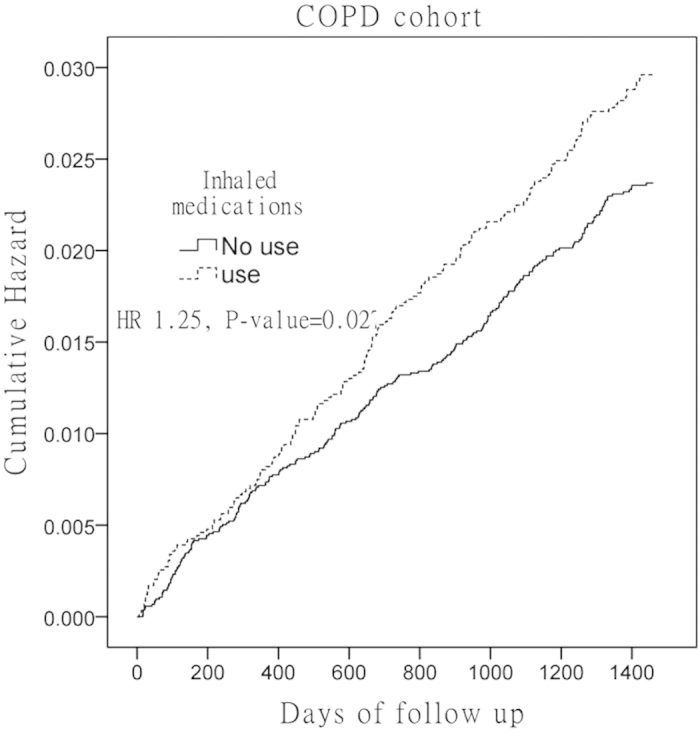
Cumulative hazard rates of hip fracture in chronic obstructive pulmonary disease (COPD) patients treated with and without Inhaled medications during the 4-year follow-up period.

Kaplan–Meier hazard curves for hip fracture in the COPD and control cohorts over the 4-year follow-up period are shown in Fig. 1. Log-rank test analysis revealed that the patients in the COPD cohort had higher risk (HR = 1.78, *P* < 0.001) than those of the patients in the non-COPD cohort (Fig. 1). Patients with COPD using inhaled medications had higher HRs (HR = 1.25, 95% CI 1.03–1.52, *P* = 0.027) for hip fracture than those of patients not using inhaled medication (Fig. 2).

## Discussion

Our large-scale population-based study revealed that patients with COPD were at an increased risk of hip fracture after we controlled for other possible contributing factors. Previous studies have revealed that most hip fractures were caused by falls and that the prevalence of falls was high among patients with COPD^20^. Therefore, we hypothesized that patients with COPD are at a risk of hip fracture. The pathogenesis of hip fractures could be explained by fall mechanisms among patients with COPD.

A previous study indicated that muscle strength, balance, and gait pattern could be intrinsic factors contributing to falls^17^,^18^,^19^. Muscle strength reduction and balance impairment can be observed in patients with COPD^24^. Moreover, Butcher *et al*. observed that patients with COPD had gait deficits^9^,^10^, which can be precipitating factors for hip fracture in patients with COPD. Adequate muscle strength and endurance are essential for maintaining postural stability and reducing postural sway^8^. Mathur *et al*. reported that patients with moderate to severe COPD had decreased muscle volume and strength and increased thigh intramuscular fat^25^. In addition to muscle strength, muscle endurance is affected by COPD. A case control study comparing the knee extensor muscle of patients with COPD and healthy participants indicated that patients with COPD showed compromised endurance and most of them could not complete the study protocol^26^. Patients with COPD had increased body sway during rest with difficulty in maintaining balance during motion of more than an arm distance^27^. Janssens *et al*. demonstrated that in patients with COPD, particularly those with inspiratory muscle weakness, postural instability was caused by decreased proprioception signal reliance on back muscles^8^,^28^. Furthermore, gait deficit is considered a contributing factor for falls in patients with COPD. Butcher *et al*. demonstrated that gait speed was lower in patients with COPD compared with healthy control participants in a 6-meter walking test^29^. In addition, a previous study noted a shorter walking distance in patients with COPD^8^. However, the direct correlation of gait speed and distance with falls remains uninvestigated. Moreover, kinematic variables of gait deficits, such as gait variability among patients with COPD, remain inadequately studied, and additional studies are warranted.

Nutrition deficit and muscle mass loss are common problems in patients with COPD^28^. Hip muscles prevent blunt injury in lateral side falls. Therefore, frail patients are less protected and more vulnerable to hip fracture^30^. Furthermore, vitamin D deficiency is associated with muscle weakness and postural instability, which increase the fall risk^31^. In addition, vitamin D deficiency is associated with secondary hyperparathyroidism, which stimulates bone resorption and increases the fracture risk^32^. A previous study showed that patients with COPD had low vitamin D levels^33^. Thus, nutrition deficit could be a factor contributing to the increased hip fracture risk among patients with COPD.

It is well established that smoking is an important risk factor for COPD and is associated with bone mineral density and increased fracture risk^34^. The adverse effect of smoking could explain the increased hip fracture risk for patients with COPD. Nicotine exposure reduces calcium absorption and influences bone modeling, resulting in low bone mineral density^35^,^36^,^37^. Therefore, smoking is a risk factor for both osteoporosis and COPD, and is a contributing factor for hip fracture among patients with COPD.

Corticosteroid use is a pathogenic factor for hip fractures among patients with COPD. It is estimated that more than 61.5% and 8.3% patients with COPD use inhaled medication and oral corticosteroids, respectively^38^,^39^. A cross-sectional study on patients with severe COPD treated with glucocorticoids with adequate calcium and vitamin levels revealed a 68% prevalence of osteoporosis and osteopenia^40^. A case control study with a large sample size revealed that patients with COPD using inhaled corticosteroids had a higher fracture risk than that of patients with COPD not using inhaled corticosteroids^41^. In addition, a systematic review and meta-analysis study revealed that a high dosage of inhaled corticosteroids is associated with an increased risk of fractures, including hip fractures, among patients with COPD. In addition to osteoporosis, respiratory and peripheral muscle weakness is caused by oral corticosteroids^42^. Long-term corticosteroid use may lead to muscle weakness, which is a pathogenic factor for hip fracture among patients with COPD. Furthermore, the study results revealed that patients with COPD using inhaled medication had a higher risk of experiencing hip fracture than that of patients not using the medication. Thus, steroids may play a crucial role in increasing the hip fracture risk among patients with COPD.

This study is the first to investigate COPD as a risk factor for hip fracture. Nevertheless, it is subject to several possible limitations. First, information on daily activities, smoking habits, body weight, and nutrition status is not recorded and quantified in the NHIRD. These factors are associated with the risk of hip fracture. Although smoking and malnutrition are common in patients with COPD, these confounders cannot be controlled completely in a large population-based study using the NHIRD. Therefore, we used a 2-stage propensity score calibration method and NHIS 2005 data for eliminating the confounding bias caused by smoking, alcohol use, and BMI. Second, COPD diagnosis and medical comorbidities were determined using ICD codes from the NHRID, and no information on the accuracy of these codes is available. To reduce the bias caused by the incorrect use of codes, we included patients who received 5 consecutive diagnoses of COPD in outpatient clinics or a primary diagnosis of osteoarthritis during hospitalization to ensure the validity of COPD diagnosis. Third, the influence of socioeconomic status on the risk of hip fracture was not presented in this study. Patients with higher socioeconomic status family generally had better medical resource and safer working environment than those families from lower socioeconomic status. COPD cohort could have lower socioeconomic status and lead them at higher risk of hip fracture. For considering this potential bias cuased by different socioeconomic status among COPD and non-COPD cohorts, we adjusted variable of income in further analysis and the data of adjusted HR was 1.60 (95% CI, 1.39–1.84, P < 0.001), which did not change the outcome of this study. Finally, the COPD severity stage and detailed medication data were not recorded in the NHIRD and can influence the hip fracture risk. To eliminate these biases, we controlled for possible confounders such as age, steroid use, and comorbidities among the study and control cohorts. However the limitations of propensity score analyses cannot eliminate all confounding definitely and no detailed data of oral glucocorticoid and osteoporosis medication usage were mentioned in this study.

## Conclusion

This population-based retrospective cohort study revealed that patients with COPD were at a 1.57-times greater risk of sustaining hip fracture. Several factors contribute to hip fracture risk, such as falls, steroid use, muscle weakness, gait deficit, impaired balance, smoking, and nutrition deficit. We recommend implementing an appropriate hip fracture prevention strategy in the public health field by using a multidisciplinary approach to improve the quality of care for patients with COPD. Additional studies investigating the effect of hip fracture risk factors in patients with COPD are warranted.

## Additional Information

**How to cite this article**: Huang, S.-W. *et al*. Chronic Obstructive Pulmonary Disease Increases the Risk of Hip Fracture: A Nationwide Population-Based Cohort Study. *Sci. Rep.* **6**, 23360; doi: 10.1038/srep23360 (2016).

## Figures and Tables

**Table 1:** Demographic characteristics and comorbid medical disorders for subjects with COPD and in the comparison cohort in LHID main database and NHIS validation database, 2004–2007.

**Variable**	**Main study**				**Validation study**			
	**Subjects with COPD N = 16239**		**Comparison Subjects N = 48717**		**Subjects with COPD N = 350**		**Comparison Subjects N = 1050**	
	**No.**	**%**	**No.**	**%**	**No.**	**%**	**No.**	**%**
Gender								
Male	10538	64.9	31614	64.9	248	70.9	744	70.9
Female	5701	35.1	17103	35.1	102	29.1	306	29.1
Age (years-old)								
51–60	3058	18.8	9174	18.8	85	24.3	255	24.3
61–70	4019	24.7	12057	24.7	76	21.7	228	21.7
>70	9162	56.4	27486	56.4	189	54.0	567	54.0
Hypertension								
Yes	8993	55.4	24032	49.3	220	62.9	549	52.3
No	7246	44.6	24685	50.7	130	37.1	501	47.7
Hyperlipidemia								
Yes	3591	22.1	10053	20.6	87	24.9	239	22.8
No	12648	77.9	38664	79.4	263	75.1	811	77.2
Stroke								
Yes	2419	14.9	5167	10.6	96	27.4	187	17.8
No	13820	85.1	43550	89.4	254	72.6	863	82.2
Diabetes								
Yes	3517	21.7	10428	21.4	96	27.4	218	20.8
No	12722	78.3	38289	78.6	254	72.6	832	79.2
Autoimmune disease								
Yes	577	3.6	1194	2.5	12	3.4	37	3.5
No	15662	96.4	47523	97.5	338	96.6	1013	96.5
Inhaled medications								
Yes	5877	36.2	1823	3.7	177	50.6	94	9.0
No	10362	63.8	46894	96.3	173	49.4	956	91.0
Nsaid								
Yes	4172	25.7	9264	19.0	191	54.6	540	51.4
No	12067	74.3	39453	81.0	159	45.4	510	48.6
Smoking								
Yes					157	44.9	368	35.0
No					193	55.1	682	65.0
Drinking								
Yes					92	26.3	315	30.0
No					258	73.7	735	70.0
BMI (SD)					23.3	(3.7)	24.0	(3.7)

Abbreviation: COPD = chronic obstructive pulmonary disease.

**Table 2:** The crude and adjusted hazard ratios for hip fracture among the sample subjects during the 4-years follow-up (N = 64956).

	**Total sample N = 64956**	**Non-COPD N = 48717**	**COPD N = 16239**
Occurrence of hip fracture, N (%)	1116 (1.7%)	715 (1.5%)	401 (2.5%)
Incidence per 100000 person-year (95% CI)	437 (435–439)	369 (367–371)	649 (644–654)
Crude HR (95% CI)	–	1.00	1.78* (1.57–2.01)
Adjusted HR (95% CI)^a^	–	1.00	1.59* (1.38–1.82)
propensity score calibration adjusted HR (95% CI)^b^	–	1.00	1.57* (1.36–1.81)

^a^Adjustment for patient’s age, sex, hypertension, stroke, hyperlipidemia and diabetes, Autoimmune and medications included Inhaled medications and Nsaid.

^b^Adjustment for patient’s age, sex, hypertension, stroke, hyperlipidemia and diabetes, Autoimmune, Inhaled medications, Nsaid and missing confounders including smoking, drinking and body mass index. *P < 0.001.

**Table 3:** Incidence, crude and adjusted hazard ratios (HRs) and 95% confidence intervals (CIs) for hip fracture during the 4-years Follow-up.

**Presence of hip fracture**	**Non-COPD N = 48717**	**COPD Patients**	
		**COPD without Inhaled medications (N = 10362)**	**COPD with Inhaled medications (N = 5877)**
follow-up period			
Yes	715	234	167
Incidence per 100,000 person-years (95% CI)	369 (342–396)	595 (519–671)	717 (606–828)
Crude HR (95% CI)	1.00	1.63* (1.40–1.89)	2.04* (1.72–2.41)
Adjusted HR^a^ (95% CI)	1.00	1.46* (1.25–1.69)	1.72* (1.45–2.04)

(*N* = 64956). Notes:

^a^Adjustments were made for age, sex, hypertension, stroke, hyperlipidemia and diabetes, Autoimmune and NSAID.

^*^Indicates p < 0.001.
